# Anisotropic shrinkage of insect air sacs revealed *in vivo* by X-ray microtomography

**DOI:** 10.1038/srep32380

**Published:** 2016-09-01

**Authors:** Liang Xu, Rongchang Chen, Guohao Du, Yiming Yang, Feixiang Wang, Biao Deng, Honglan Xie, Tiqiao Xiao

**Affiliations:** 1 Shanghai Institute of Applied Physics, Chinese Academy of Sciences, Shanghai 201204, China; 2 University of Chinese Academy of Sciences, Beijing 100049, China

## Abstract

Air sacs are thought to be the bellows for insect respiration. However, their exact mechanism of action as a bellows remains unclear. A direct way to investigate this problem is *in vivo* observation of the changes in their three-dimensional structures. Therefore, four-dimensional X-ray phase contrast microtomography is employed to solve this puzzle. Quantitative analysis of three-dimensional image series reveals that the compression of the air sac during respiration in bell crickets exhibits obvious anisotropic characteristics both longitudinally and transversely. Volumetric changes of the tracheal trunks in the prothorax further strengthen the evidence of this finding. As a result, we conclude that the shrinkage and expansion of the insect air sac is anisotropic, contrary to the hypothesis of isotropy, thereby providing new knowledge for further research on the insect respiratory system.

Insects are among the most diverse groups of animals on the earth, comprising approximately 5.5 million described species and representing more than half of all known living organisms^1^. Instead of lungs, insect respiration uses a system of internal tubes and air sacs through which gases either diffuse or are actively pumped, delivering oxygen directly to tissues. The branching tracheal tubes are formed from invaginations of the entire integument of the cuticle. Gaseous exchange between the external atmosphere and internal tissues occurs through openings called spiracles, which usually have closable valves. The flexible air sacs in insects are enlarged tracheae and lack the normal exocuticle and taenidia, which are thought to be bellows to aid convective airflow^2^,^3^. Traditionally, the study of respiration in insects was primarily limited to external gaseous exchange through spiracles. Such studies have provided valuable insights into insect respiratory physiology^4^,^5^,^6^,^7^,^8^ but little knowledge about what happens to the air inside the respiratory system and the mechanism by which the air sacs act as a bellows. In 2003, Westneat *et al*. visualized the internal tracheal system of live insects for the first time using synchrotron radiation X-ray radiography^9^. Combined with synchronous measurements of the patterns of carbon dioxide emission and active ventilation movements, profound insights into convection and diffusion in the respiratory process were obtained^10^,^11^,^12^,^13^. In previous work, the shrinkage of the air sac has usually been assumed to be isotropic^14^,^15^. Under this hypothesis, the ventilation frequency and the compression ratio of the air sac could be inferred from the time sequences of the two-dimensional projections using X-ray radiography. However, this hypothesis has never been validated experimentally by directly observing the three-dimensional evolution of the air sac *in vivo*. Momose *et al*. used Talbot interferometry to successfully visualize a drastic change in the respiratory tract of a living worm^16^. However, the high radiation dose due to the white beam prevented the application of this method for *in vivo* imaging of live insects. Another remarkable *in vivo* time-resolved microtomography was acquired by Walker *et al*. in which the steering muscles in the thorax of the blowfly were investigated^17^.

In this study, dynamic X-ray phase contrast microtomography, a type of four-dimensional X-ray imaging, is employed to observe the dynamic evolution of the three-dimensional structure of insect air sacs *in vivo* and *in operando*. Monochromatic X-ray beams are employed to ensure low radiation doses and high image contrast for the *in vivo* imaging of live insects. Quantitative analysis is also conducted to elucidate the respiratory dynamics of insects.

## Results and Discussion

**Figure 1:**
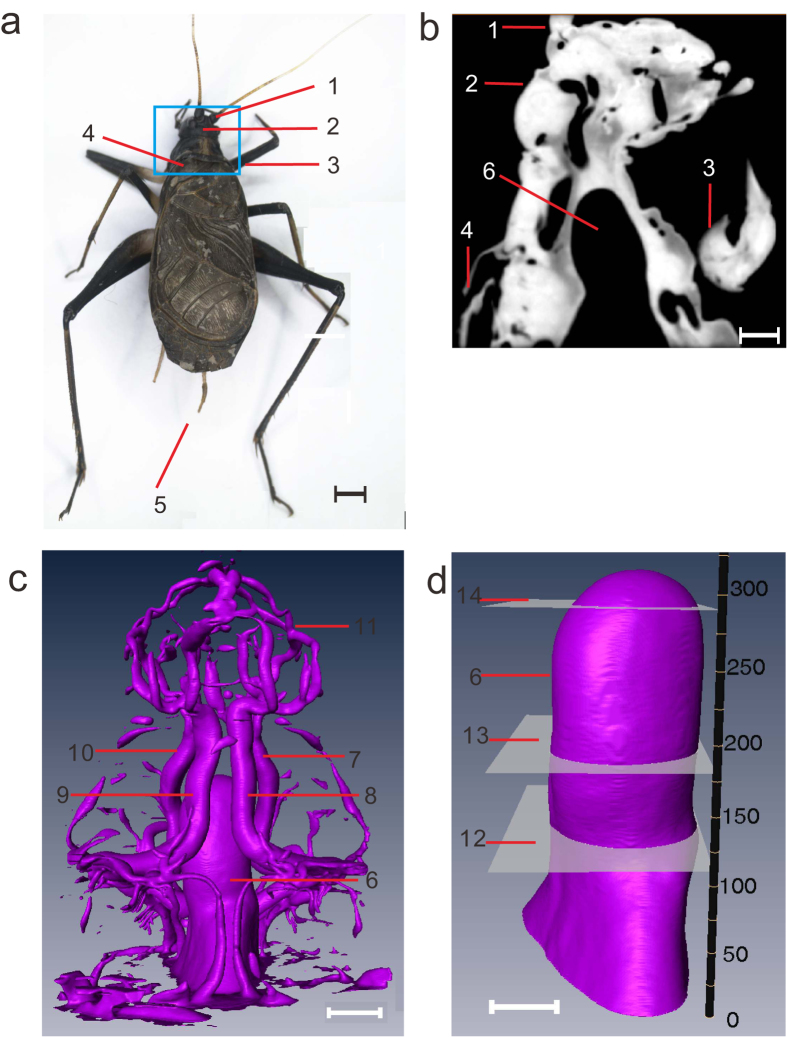
Picture of the bell cricket and its corresponding *in vivo* images. (**a**) The photograph of the bell cricket used in experiments. The region of interest (ROI) for the imaging is noted with a blue rectangle frame; length of the scale bar is 2 mm. (**b**) The longitudinal section in the middle of the ROI reconstructed from three-dimensional microtomography, where the corresponding structures in (**a**) are labelled; length of the scale bar is 500 μm. (**c**) The tracheae extracted from the ROI, with the back of the bell cricket to the observers; length of the scale bar is 600 μm. (**d**) The air sac extracted individually from (**c**). A Z-axis is given to locate the position of each slice; length of the scale bar is 380 μm. 1—antennae; 2—head; 3—legs on the prothorax; 4—wings; 5—cerci; 6—air sac; 7—tracheal trunk 1 in the prothorax; 8—tracheal trunk 2 in the prothorax; 9—tracheal trunk 3 in the prothorax; 10—tracheal trunk 4 in the prothorax; 11—tracheae in the head; 12—the 110*th* slice of the air sac; 13—the 180*th* slice of the air sac; and 14—the 290*th* slice of the air sac.

A typical insect species, the bell cricket (*Meloimorpha japonica*; Haan, 1842), was selected as the sample. The size of the bell cricket is approximately 6 mm × 17 mm. A photograph of the bell cricket, with the region of interest (ROI) noted, is shown in Fig. 1a. From Fig. 1a, we can clearly see antennae, head, legs, wings and cerci. The reconstructed longitudinal section in the middle of the ROI is shown in Fig. 1b, and the dark regions inside the bell cricket are the profiles of the tracheae. Thanks to the high contrast between air and tissues, we can clearly separate the tracheae from other tissues (Supplementary Movie 1). The tracheal tubes spread throughout the whole body, which is consistent with the findings of previous studies. An overview of the tracheal system is shown in Fig. 1c. The upper portion is the reticular tracheae in the head, while the lower portion is the dendriform tracheae in the prothorax. Four main tracheal trunks connect the respiratory system in the head and the prothorax. Because the bell cricket does not experience strenuous exercise, only the air sac shows obvious rhythmic compression; other smaller tubes are nearly static. This result can be observed clearly in Supplementary Movies 2,3,4,5, which show four viewing angles. As shown in Fig. 1d, the air sac is extracted from Fig. 1c to examine its dynamics more distinctly, and a Z-axis is inserted to locate the position of each slice. Supplementary Movie 6 shows the compression of the air sac separately, in which different portions of the air sac show various compression situations.

**Figure 2:**
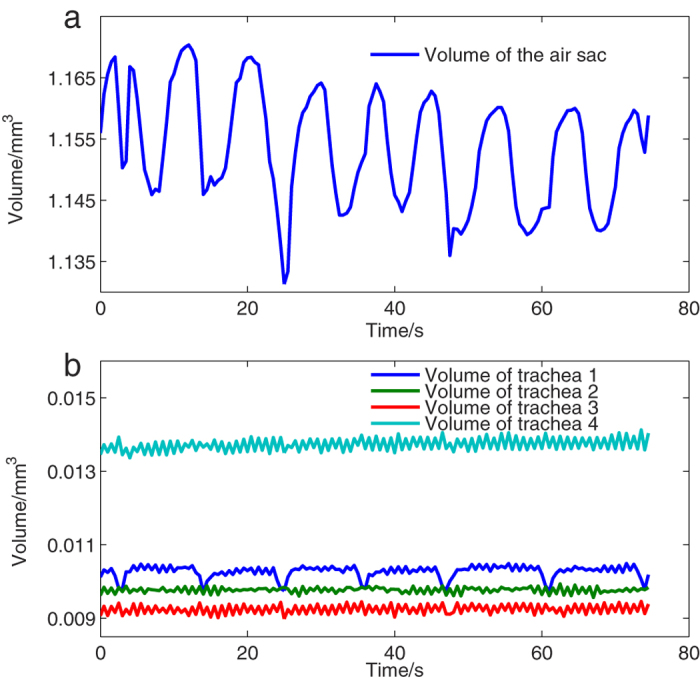
Quantitative volumetric changes. (**a**) Volume of the air sac as a function of time. In the first few seconds, the volumetric changes show an erratic pattern. Next, the volumetric changes come into a steady state and represent a periodic pattern within the seven full periods captured. The periods have small fluctuations with an average of 8.7 s. (**b**) Volumes of tracheal trunk 1–4 in the prothorax as a function of time. The volume of trachea 1 has a periodic change with a period of 11.5 s, while others show irregular change.

Volumetric changes of the air sac are direct indicators of the respiratory process and provide information on the frequency and strength of respiration. Figure 2a shows the volume of the air sac as a function of time. An obvious periodic change is shown, and seven complete periods are captured during the experiments. In the first few seconds, the plot shows an erratic pattern, which suggests that the insect cannot acclimate to the new status at the beginning of the experiments. The volumetric change then proceeds into a steady periodic process. The peaks experience a slight decrease during the first 50 s and then tend to be stable. The duration of the two adjacent peaks or valleys is defined as the respiratory period, and the difference between the peak and the adjacent valley is defined as the air input or output. The periods have small fluctuations with an average of 8.7 s, despite variability in the values of peaks or valleys. This finding suggests that the bell cricket experienced an undisturbed respiratory state during the experiments. The average air input is 0.019 mm^3^ and the output is 0.021 mm^3^ during a single respiratory process. These results indicate that the air input and output remain balanced to some extent. The upper portion of the air sac experiences a more drastic compression, and different parts of the air sac represent diverse compression directions and frequencies, which is inconsistent with the previous hypothesis of isotropic shrinkage (Supplementary Movie 6). Additional evidence is needed to confirm this finding. The volumes of four thoracic tracheae at different time points are calculated separately and shown in Fig. 2b, in which volumetric changes of four main tracheal trunks in the prothorax are also obviously different. Volumes of trachea 2–4 fluctuate slightly, while that of trachea 1 has a regular mode with a period of 11.5 s. This slight volumetric change in the tracheae differs from that described in the work of Westneat *et al*.^9^, which may result from the constraint of limb movements in our experiments. To elaborate the anisotropic shrinkage of the air sac quantitatively, we analysed compression of the air sac both longitudinally and transversely below.

**Figure 3:**
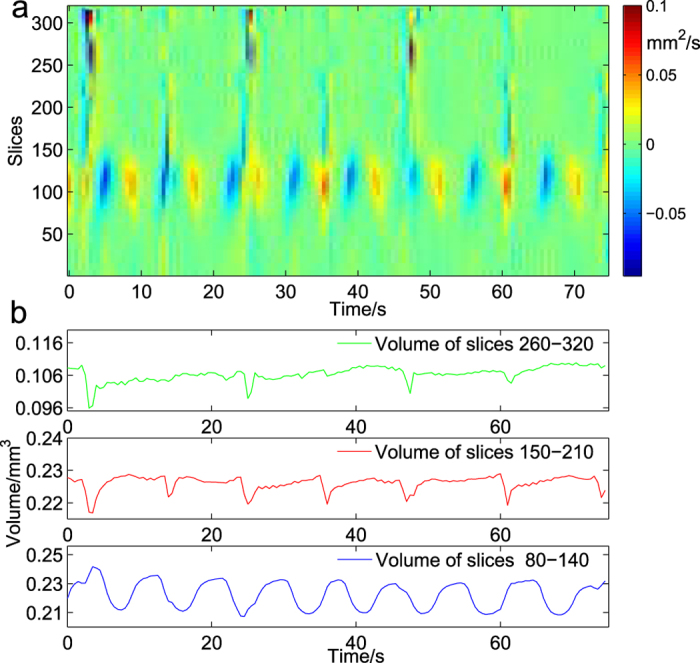
The longitudinal respiratory pattern of the air sac. (**a**) The changing rate of every cross-sectional area of the air sac. Each pixel in this image gives the changing rate of the specified cross-sectional area at a specified time point. The X-axis represents the time, and the Y-axis represents the slice number of the air sac. The colour represents the changing rate of the cross-sectional area. (**b**) The segmented volumes of the air sac as a function of time. The volumetric change of slices 80–140 is in sinusoidal form, with an average period of 8.5 s. The volumetric change of slices 150–210 reaches a local minimum with a period of 11.8 s, and the volumetric change of slices 260–320 reaches a local minimum with a period of 22.5 s. The volumes between each two local minima change slightly.

Shown in Fig. 3a is the changing rate of the cross-sectional area of the air sac along the longitudinal direction (the Z-axis direction shown in Fig. 1d). The X-axis and Y-axis represent time and slice number, respectively. The colours represent changing rates; the red colour represents expansion, and the blue colour represents shrinkage. There are three different changing patterns around the 110*th*, 180*th* and 290*th* slice of the air sac. Near the 110*th* slice, the colours exhibit a periodic pattern. We take the blue bulk at the 30th second as an example. As the time passes (from left to right along the X-axis), the blue bulk goes higher, which means that the compression of the air sac occurs from the lower cross-section to the upper one. The red and yellow regions show the reverse trend, which means that the expansive direction is inverse. Around the 180*th* and 290*th* slices, the changes in colours along the X-axis are similar. As shown in Fig. 3a, at the 25*th* second, a short duration of the blue colour is next to a short duration of the red colour, followed by a longer duration of the green colour. These changes in colours indicate that the portions around the 180*th* and 290*th* slice of the air sac experience short-lived compression every once in a while. These results inspired us to investigate and compare the segmented volumetric changes of the air sac. During the quantitative analysis, the segmented volume is defined as the sum of the voxels in the selected slices. According to the changing rate of the cross-sectional area in Fig. 3a, we selected blocks of slices 80–140, 150–210 and 260–320 as the segmented volume of the air sac. These results are shown in Fig. 3b. The average changing period of the volume around the 110*th* slice is 8.5 s, which matches the respiratory period of the entire air sac. The period of volumetric changes around the 180*th* is 11.8 s, similar to the period of trachea 1 in the prothorax, which means that trachea 1 is possibly driven by this portion of the air sac. In addition, the period of volumetric changes around the 290*th* is 22.5 s, suggesting that the portion at the end of the air sac acts passively and is far from the driven point of the compression. As shown in Fig. 3b, the values of these three segmented volumes synchronously reach a minimum at the 25*th* second, which may explain the drastic compression shown in Fig. 2a at that moment. The different compression behaviour along the longitudinal direction of the air sac is defined as longitudinal anisotropic shrinkage. Aboelkassem and Staples found that contractions moving on the tracheal wall with non-zero phase lags with respect to each other could push the gas along a desired direction^18^. In our experiments, considering the volumetric changes of the three segmented volumes together, we find that three parts of the air sac have different periods of compression. The non-synchronous compression possibly pushes the air from the air sac to tracheae. According to the quantitative analysis of the three-dimensional structure of the air sac, the average segmented volumetric compression ratios near the 110*th*, 180*th* and 290*th* slices are 4.8%, 1.8% and 3.1%, respectively. These results further strengthen the evidence for longitudinal anisotropic shrinkage. In Wasserthal’s work, flying blowflies generated a unidirectional ventilatory airflow, and the oxygen levels within thoracic air sacs nearly reached ambient levels during full flight^19^. This suggests that the longitudinal anisotropic shrinkage of the air sac also helps to supply sufficient oxygen for the insect’s strenuous activities.

**Figure 4:**
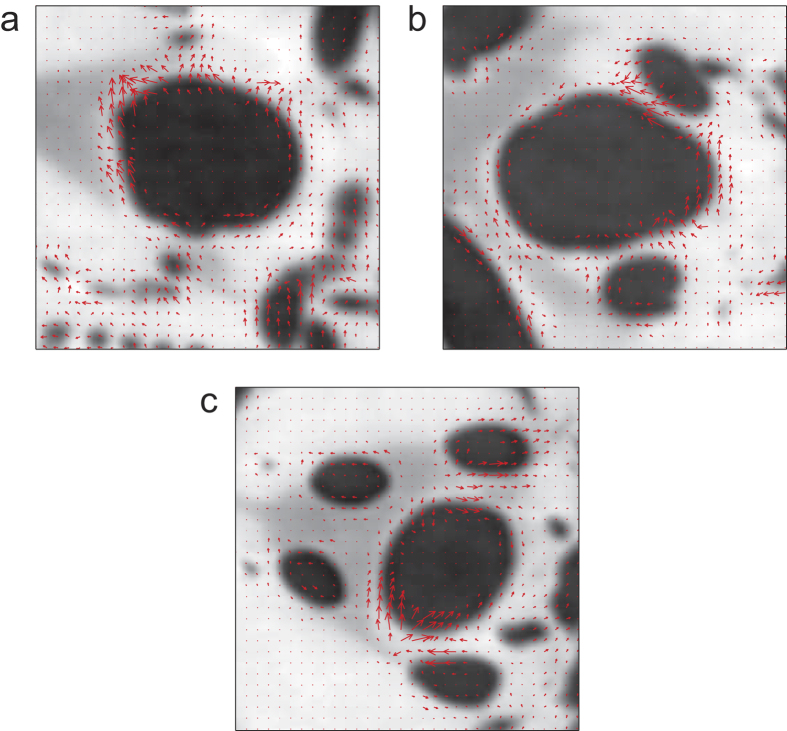
The transverse velocity fields at the 110*th*, 180*th* and 290*th* slices of the air sac at the moment of the 25*th* second from the start of the experiment. The maximum black regions in the middle of these three slices are the transverse sections of the air sac. The red arrow represents the direction of motion; the length represents the value of speed. (**a**) The transverse velocity fields of the 110*th* slice. The velocity vectors at the top left corner of the air sac show an outward trend, while velocity vectors at the bottom right corner are nearly zero. The other portions of this section show an expanding process with moderate speed. (**b**) The transverse velocity fields of the 180*th* slice. The compression portion is located at the bottom right corner, and the inflation portion is located at the top right corner. Other velocity vectors are nearly zero. (**c**) The transverse velocity fields of the 290*th* slice. The left edge of the air sac exhibits a shrinking trend, while other portions are nearly still.

Based on the volume analysis along the longitudinal direction of the air sac, characteristics of the longitudinal anisotropic shrinkage are investigated. How the compression performs at the cross-section of the air sac remains unknown. Three typical slices (the 110*th*, 180*th* and 290*th* slice) are selected to implement such an investigation. The vector field of the velocity for the edge profile of the air sac is used to characterize the compression behaviour at a cross-section. As shown in Fig. 4, the velocity fields at the 110*th*, 180*th* and 290*th* slices are given, which show the action of air sac at the moment of the 25*th* second. For reference, the velocity fields of the muscle around the air sac are also provided. The arrow represents the direction of motion; the length represents the value of speed. For further reference, the full movies of the compression of these three slices are in Supplementary Movies 7,8,9. Figure 4a shows the velocity field at the 110*th* slice of the air sac. The velocity vectors at the top left corner of the air sac (the black region in the middle) show an outward tendency, which means that this portion experiences an obvious expanding process compared to the other portions. The adjacent muscle also experiences a similar pattern of movement. However, velocity vectors at the bottom right corner are nearly zero, which implies that this portion remains almost still at the same time. The other portions at this section show an expanding process with moderate speed. In the 180*th* slice shown in Fig. 4b, the shrinking portion is located at the bottom right corner of the air sac, while the expanding portion is located at the top right corner. There is no muscle adjacent to these portions; thus, compression and inflation may result from changes in the internal pressure. On the whole, the right portion has an upward tendency, while the left portion remains nearly still. As shown in Fig. 4c for the 290*th* slice, the left edge of the air sac exhibits a shrinking trend, while other portions are nearly still. As a result, the compression behaviours of different edge profiles of the same cross-section of the air sac are also different. We define this phenomenon as transverse anisotropic shrinkage. This non-synchronous compression at the same cross-section of the air sac might also help ventilate the air in tracheae. We observe the compression of the air sac, while the action of the muscles is not revealed. There are two possible reasons for this observation. First, the density differences between muscles are too small to be resolved. Second, the action of the muscles may be a pulse, and the time duration is too short to be caught by the dynamic CT at a time scale of 500 ms.

Air sacs are thought to be the bellows for the insect respiratory system, but their exact mechanism of action as a bellows remains unclear. One method to investigate the authentic air-driving behaviour of the air sac is the *in vivo* observation of the changes in their three-dimensional structure. In this study, four-dimensional X-ray phase contrast microtomography is employed to solve this puzzle. Quantitative analysis of the three-dimensional image series reveals that the compression of the air sac in bell crickets shows obvious anisotropic characteristics both longitudinally and transversely. Compression behaviour of the thoracic trachea 1–4 was also analysed; tracheae 1 experiences a periodic compression, and the others change slightly. Based on the experimental results and related analysis, we conclude that the shrinkage and expansion of the insect’s air sac is anisotropic, which is likely what drives air to the tracheae, resembling a bellows. These results may provide a deeper understanding of the dynamics of insect respiration.

## Methods

### Sample preparation

One live mature bell cricket was used and settled in a ventilated plastic container at a normoxic state and at room temperature. The plastic container was used to ensure that movements of the cricket were negligible during the imaging process. Before the experiments, the cricket stayed still in the container for half an hour to adapt to the new surroundings.

### X-ray microtomography

The experiments were conducted at BL13W at Shanghai Synchrotron Radiation Facility^20^. The white beam from a wiggler source was monochromized via a Si (111) or Si (311) double crystal monochromator cooled by liquid nitrogen. The energy range of output X-rays was 8–72.5 keV, with an energy resolution less than 0.5%. A composite slit was installed to restrict the size of the monochromatic beam. An ionization chamber was set downstream to monitor the flux during the experiments. The monochromatic beam has a full field of view of 45 mm (H) × 5 mm (V). The critical elements of the dynamic X-ray microtomography (SR-μCT) system are a sCMOS based fast detector (Hamamatsu ORCA-Flash4.0, maximum frame-rate 1000 Hz with a pixel size of 6.5 μm) and an air bearing rotation stage (PI miCos, UPR-120 AIR, maximum speed 360 °/sec). To obtain optimal results, the experimental parameters were carefully chosen. The photon energy was set to 14 keV, the sample-to-detector distance was set to 50 cm, the region of interest was 2048 × 700 pixels (horizontal × vertical), the exposure time of one single projection was 2 ms and the rotation speed was 360 °/sec. Under these parameters, the acquisition time of one data set was 500 ms, including 136 projections over a full 180° range. The experiment lasted 75 s, and 150 data sets were obtained. After image reconstruction, we obtained the three-dimensional dynamical structures of the air sac at 150 successive time points.

### Data analysis and quantification

Data were processed using the PITRE software package for phase-sensitive X-ray image processing and tomography reconstruction^21^. The reconstructed tomograms were 8-bit grey-scale, in which the materials with the highest refractive index were displayed as white and the lowest refractive index as black. The three-dimensional reconstructions were made by Amira 5.4 (FEI VSG, France), where the three-dimensional images were displayed as stacks of 2D image slices. Next, the air sac was extracted from these reconstructed images using the seeded region growing algorithm^22^, and the AVI format movies were created with the free software, ImageJ. The areas of cross-section slices of the air sac and the volume of the air sac were calculated by counting the number of pixels that they occupied. The compression ratio of the volume of the air sacs during a respiratory cycle was calculated using the formula *(volume*_*max*_ − *volume*_*min*_*)/(volume*_*max*_ + *volume*_*min*_). Taking the Fig. 2a as an example, the plot is in sinusoidal form with seven complete periods. If we focus on a period, *volume*_*max*_ means the peak value and *volume*_*min*_ means the valley value. The changing rate of the cross-sectional area of the air sac for each slice was obtained by calculating differences in the cross-sectional area of slices of neighbouring time points at the same height.

### Velocity field calculation

To calculate the velocity field, an optical flow method was used^23^. The unknown 2D velocity vector **u** = (*u, v*) for each pixel position **x** = (*x, y*) between two successive images *I* (**x**, *t*) and *I* (**x**, *t* + *Δt*) was determined by polynomial expansion. The idea of polynomial expansion is to approximate some neighbourhood of each pixel with quadratic polynomials, given by *I* (**x**, *t*) = **x**^T^**A_1_x** + **b**_1_^T^**x** + *c*_*1*_, where **A**_1_ is a symmetric matrix, **b_1_** is a vector and *c*_*1*_ is a scalar. At a successive time point, the image has a displacement **d**. The image can then be expressed in the local coordinate system, *I* (**x**, *t* + *Δt*) = **x**^T^**A_2_x** + **b**_2_^T^**x** + *c*_*2*_. Under the assumption of constant image brightness, we obtain *I* (**x**, *t* + *Δt*) = *I* (**x** − **d**, *t*), and then **d** = −**A**_1_^−1^(**b**_2_ − **b**_1_)/2. Combined with another assumption that the displacement field varies little, we can obtain a more practical result **d** = (∑*w***A**^T^**A**)^−1^(∑*w***A**^T^*Δ***b**), where *w* is a weight function for the points in the neighbourhood, **A** = (**A **_1_+ **A**_2_)/2 and *Δ***b** = (**b**_2_ − **b**_1_)/2. The velocity field can then be calculated by **u** = **d**/*Δt*.

## Additional Information

**How to cite this article**: Xu, L. *et al*. Anisotropic shrinkage of insect air sacs revealed *in vivo* by X-ray microtomography. *Sci. Rep.* **6**, 32380; doi: 10.1038/srep32380 (2016).

## Supplementary Material

Supplementary Movie 1

Supplementary Movie 2

Supplementary Movie 3

Supplementary Movie 4

Supplementary Movie 5

Supplementary Movie 6

Supplementary Movie 7

Supplementary Movie 8

Supplementary Movie 9

Supplementary Information
