# Supplementary material for: Anisotropic shrinkage of insect air sacs revealed in vivo by X-ray microtomography
Source: Sci Rep. 2016 Sep 1;6:32380. doi: 10.1038/srep32380 (PMC5007674; doi:10.1038/srep32380)
Supplement: Supplementary Information [file srep32380-s10.pdf]

Supplementary Materials for

Anisotropic shrinkage of insect air sacs revealed *in vivo* by X-ray microtomography

L. Xu, R. Chen, G. Du, Y. Yang, F. Wang, B. Deng, H. Xie and T. Xiao*.

***Correspondence to:** xiaotiqiao@sinap.ac.cn

Supplementary Movie 1 The presentation of separating respiratory structures from other tissues. The tracheal structures are extracted according to pixel values. From the separating process, we can see that the tracheal tubes spread throughout the whole body.

Supplementary Movie 2 The respiratory process shown from viewing angle of 0 degrees. Movie 2 shows the structural change of the tracheal structures. The air sac is in the middle of the structures and a red arrow points to the air sac. The air sac represents rhythmic compression; other smaller tubes are nearly static. We can see the lower half of the air sac compresses and inflates alternately, while the upper half of the air sac provides a pulsating compression. The time scale of this movie is 1 second to 5 seconds and this movie corresponds to data acquired from 13 s to 38 s in real time.

Supplementary Movie 3 The respiratory process shown from viewing angle of 90 degrees. This movie validates the conclusion of movie 2 from viewing angle of 90 degrees. The time scale of this movie is 1 second to 5 seconds and this movie corresponds to data acquired from 13 s to 38 s in real time.

Supplementary Movie 4 The respiratory process shown from viewing angle of 180 degrees. This movie validates the conclusion of movie 2 from viewing angle of 180 degrees. The time scale of this movie is 1 second to 5 seconds and this movie corresponds to data acquired from 13 s to 38 s in real time.

Supplementary Movie 5 The respiratory process shown from viewing angle of 270

degrees. This movie validates the conclusion of movie 2 from viewing angle of 270 degrees. The time scale of this movie is 1 second to 5 seconds and this movie corresponds to data acquired from 13 s to 38 s in real time.

Supplementary Movie 6 Shrinkage of the air sac during respiration. The air sac is extracted from the tracheal structures to examine its dynamics more distinctly. The lower part of the air sac experiences sinusoidal evolution; however, the upper part presents a pulsating change with a longer period. This compression pattern is interesting. The time scale of this movie is 1 second to 2.5 seconds and this movie corresponds to data acquired from 0 s to 75 s in real time.

Supplementary Movie 7 The shrinkage of the air sac at the 110*th* slice. The 110*th* slice of air sac is the black region in the middle. From this movie, we can see the left edge profile of the air sac at the 110*th* slice shrinks and expands continuously, while the right one remains nearly still. The time scale of this movie is 1 second to 5 seconds and this movie corresponds to data acquired from 0 s to 75 s in real time.

Supplementary Movie 8 The shrinkage of the air sac at the 180*th* slice. From this movie, we can see the bottom left corner of the air sac at the 180*th* slice remains nearly still, while other portions shrink and expand suddenly. The time scale of this movie is 1 second to 5 seconds and this movie corresponds to data acquired from 0 s to 75 s in real time.

Supplementary Movie 9 The shrinkage of the air sac at the 290*th* slice. From this movie, we can see the right edge profile of the air sac at the 290*th* slice remains nearly still, while other portions present pulsating shrinkage. The time scale of this movie is 1 second to 5 seconds and this movie corresponds to data acquired from 0 s to 75 s in real time.
